# Acute ECG ST-segment elevation mimicking myocardial infarction in a patient with pulmonary embolism

**DOI:** 10.1186/1476-7120-8-50

**Published:** 2010-11-24

**Authors:** Tomaž Goslar, Matej Podbregar

**Affiliations:** 1 Clinical Department for Internal Intensive Care, University Medical Center Ljubljana, Slovenia

## Abstract

Pulmonary embolism is a common cardiovascular emergency, but it is still often misdiagnosed due to its unspecific clinical symptoms. Elevated troponin concentrations are associated with greater morbidity and mortality in patients with pulmonary embolism. Right ventricular ischemia due to increased right ventricular afterload is believed to be underlying mechanism of elevated troponin values in acute pulmonary embolism, but a paradoxical coronary artery embolism through opened intra-artrial communication is another possible explanation as shown in our case report.

## Background

Pulmonary embolism is a common cardiovascular emergency, but it is still often misdiagnosed due to its unspecific clinical symptoms, so various models have been developed to help its diagnosis [1]. Dyspnoea, tachypnoea and chest pain are presenting symptoms in more then 90% of cases [2]. Electrocardiographic (ECG) changes are very unspecific and range from most common sinus tachycardia, rightward shift in QRS axis, complete or incomplete right bundle branch block, precordial T wave inversion, S1Q3T3 pattern and more uncommon ST segment elevation [3,4]. A few case reports describe ECG presentation of pulmonary embolism as ST segment elevation in precordial leads, but exact mechanism is still unclear [4-6].

Elevated troponin concentrations are associated with greater morbidity and mortality in patients with pulmonary embolism [2,7,8]. Right ventricular ischemia due to increased right ventricular afterload is believed to be underlying mechanism of elevated troponin values in acute pulmonary embolism, but a paradoxical coronary artery embolism through patent foramen ovale (PFO) or other right-to-left atrial communication is another possible explanation at least in some cases [6,9-11].

## Case presentation

57-year old man presented to emergency department of regional hospital with chest pain, which started after walking in the morning. Dull pain was localized in the middle of his chest and was accompanied with nausea, fatigue and difficult breathing.

He had no history of heart problems, but was a former smoker and had elevated cholesterol levels. During last summer, he noticed varicose veins on right groin and was wearing elastic bandage. He had no history of cardiovascular disease, took no medications, had no known allergies and was in good physical condition. His father died at the age of 80 due to cerebrovascular insult and his mother had pulmonary embolism at the age of 74.

Since chest pain, dyspnea and fatigue didn't subside he called an ambulance. Upon arrival to emergency department he was pale and hypotensive (102/60 mmHg), heart rate was 100 b.p.m. and respiratory rate 22 breaths/min. Oxygen saturation was 95% while breathing 100% oxygen. His body temperature was 36.6 C°. Beside varicose veins on right groin, his physical examination was unremarkable.

**Figure 1:**
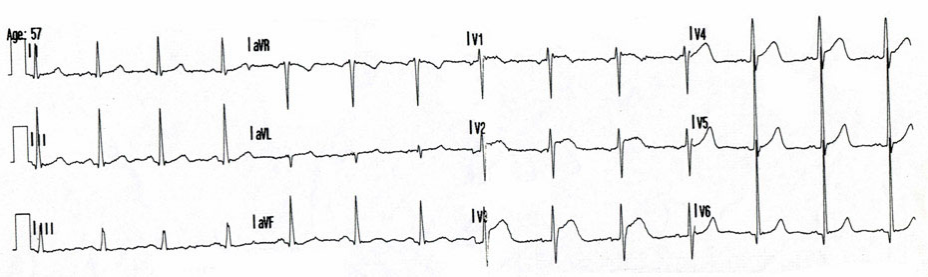
**12-lead ECG of patient with suspected STEMI recorded in regional hospital**.

On 12-lead ECG there was sinus rhythm with incomplete right bundle branch block, ST segment elevations up to 3 mm in precordial leads V1-V4 and negative T wave in lead III (Figure 1).

On the basis of ECG changes and clinical presentation, acute ST segment elevation myocardial infarction (STEMI) was suspected. After receiving aspirin 500 mg, morphine 5 mg i.v., thiethylperazine, unfractionated heparin 5000 IU i.v. and nitroglycerin sublingually, he was immediately transferred to our University medical center for primary coronary intervention (PCI). In case of STEMI, quick referral from local hospital or directly from the field, to catheterisation laboratory is a standard of care in Slovenia. That is why no further diagnostic procedures, which would delay transport to PCI were performed in regional hospital.

**Figure 2:**
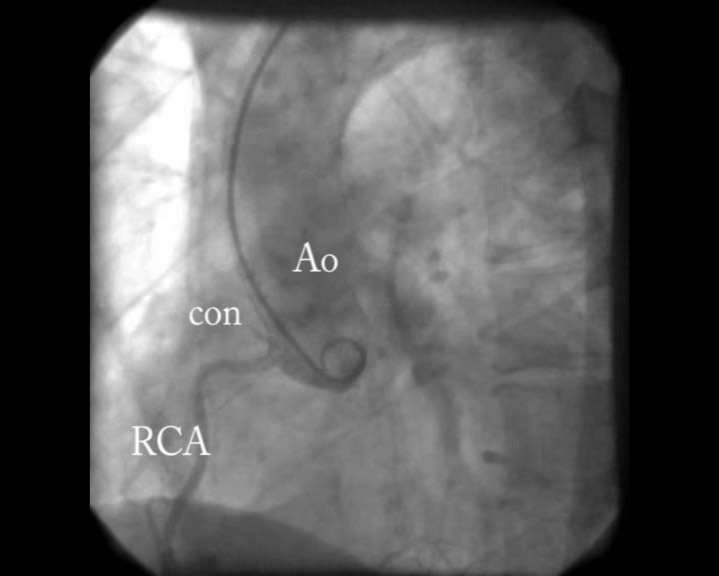
**Coronaro-angiography showing occluded conus artery**. Ao- aorta, con- conus artery, RCA- right coronary artery

The coronaroangiography reveled normal ascending aorta, coronary arteries without atherosclerotic lesions and acute occlusion, however there was a small separate conus artery arising from aortic root with embolic occlusion (Figure 2, additional file 1). The affected artery was too small to attempt PCI.

On admission to medical intensive care unit (ICU) his first blood tests were available. His white blood cell count was slightly elevated (13.4 10^9^/L), so were troponin I (0.128 μg/l - cut off 0.1 μg/l) and D-dimmer (1172 μg/l - cut off 350 μg/l). 4 hours after the onset of symptoms brain pro-BNP concentration was still normal (332.8 ng/l - cut off 500 ng/L). Lactate concentration was not elevated. Arterial blood gas analysis while breathing 6 liters of oxygen via bi-nasal catheter showed pH 7.41, carbon dioxide pressure of 4.3 kPa, oxygen pressure of 17.3 kPa, bicarbonate concentration of 19.9 mM and BE -3.7 mM.

Transthoracic echocardiography reviled good left ventricular systolic function without segmental contraction defects. Right cardiac chambers were enlarged, paradoxical movement of interventricular septum was noted but contractility of right ventricle was not impaired and right heart pressure was estimated to be 40 mmHg + central venous pressure.

**Figure 3:**
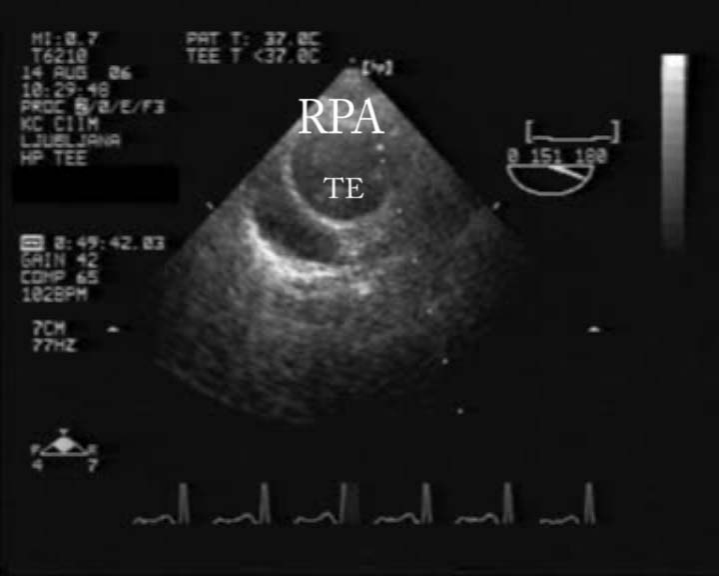
**Transesophageal echocardiography showing trombemboli in right pulmonary artery**. Tromboemboli (TE) in right pulmonary artery (RPA)

**Figure 4:**
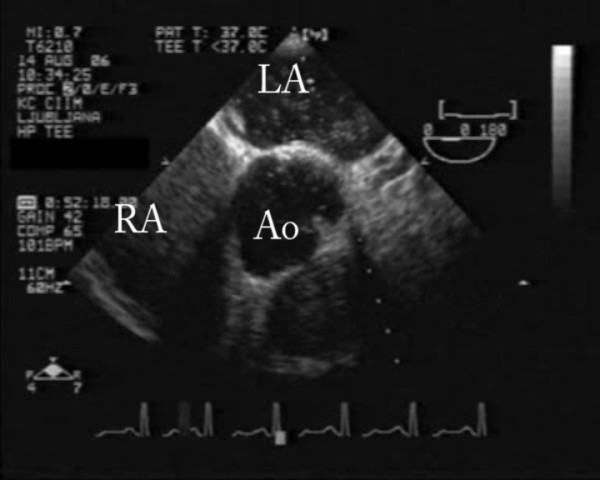
**Transesophageal echocardiography showing right to left intra-atrial shunt after contrast application**. Ao- aorta, LA- left atria, RA- right atria

With bedside transesophageal echocardiography pulmonary embolism was confirmed; thrombi were seen in both pulmonary arteries (Figure 3, additional file 2); right to left intra-atrial communication was diagnosed after contrast application (Figure 4, additional file 3).

At the time of diagnosis of pulmonary embolism he was hemodynamically stable. He received continuous infusion of unfractionated heparin and was soon transferred to step down telemetry unit. Maximal troponin I value of 3.08 μg/l was reached 24 h after admission. ST segment elevations of precordial leads returned to baseline and inverse T waves appeared on leads V1-V3. Ultrasound of lower extremity veins reveled right popliteal vein thrombosis. In a few days heparin infusion was substituted for warfarin and he was discharged.

## Discussion

In our case, patient presented with electrocardiographic picture of acute ST-elevation in precordial leads and suspected myocardial infarction. Coronaroangiography reveled embolisation in conus coronary artery, which could not fully explain patients symptoms and the embolus origin. In the presence of pulmonary embolism with proven intra-atrial communication and right to left shunt, paradoxical embolism to conus coronary artery seams the most obvious explanation for ST segment elevation in precordial leads V1-V4.

Studies have shown that acute right ventricular hypertension and consequently increased right ventricular afterload produced by pulmonary artery occlusion result in right ventricular failure. Acute right ventricular hypertension decreases coronary driving pressure and associated with increased demand result in right ventricular failure, ischemia and subsequent pump failure [10,11]. Elevated troponin values due to right ventricular ischemia and failure are associated with increased morbidity and mortality in patients with pulmonary embolism [7-9,12,13].

ST elevation in precordial leads in cases of pulmonary embolism could be a result of ischemia of acutely pressure-overloaded right ventricle or ischemia due to insufficient coronary perfusion due to previous coronary artery stenosis or paradoxical embolism with coronary artery occlusion; all or different combinations of these mechanisms could be present. However, only few cases of pulmonary embolism presenting with ST elevation in precordial leads have been published, but non of these could fully explain mechanism of ECG changes. In available literature we only found one published case of pulmonary embolism with paradoxical embolism to coronary artery, which presented as non-ST elevation changes in ECG.

Wilson et all reports a case of 57 year old man presenting with syncopal episode, followed by midsternal chest pain and shortness of breath. ECG reveled right bundle branch block (RBBB), substantial ST segment elevation and pathologic Q waves in the anterior and inferior leads consistent with acute anteroseptal myocardial infarction. Cardiac catheterization however showed only moderate atherosclerotic coronary artery disease without acute oclusion. Ventilation-perfusion scan was highly suggestive of pulmonary embolism. Patient had elevated troponin I levels, but ST segment elevations resolved without occurrence of pathological Q waves [5].

Lin et all describes a case of 35 year old man with right leg pain and collapse, followed by severe chest pain and dyspnea. ECG showed normal sinus with incomplete RBBB, ST elevation s in leads V1-V4, S waves in I, V5-V6 and Q wave in lead III also suggestive of myocardial infarction. In one hour after administration of low molecular weight heparin administration ST segment elevations resolved back to normal. Coronarography showed normal coronary arteries. Pulmonary embolism was confirmed with pulmonary angiography. There was no rise in cardiac enzymes [14].

Falterman et all describes a case of 62 year old man presenting with dispnea followed by syncopal episode. First ECG showed sinus rhythm, left anterior fascicular block and T wave inversion in leads V1-V3. While waiting for hospital admission another syncopal episode followed. Second ECG showed sinus tachycardia, incomplete RBBB, S wave in lead I, Q wave in lead III and ST segment elevation in leads V1-V4, consistent with acute anteroseptal myocardial infarction. Shortly after patient went into cardiopulmonary arrest due to ventricular fibrillation. Despite prolonged resuscitation the patient died. An autopsy demonstrated a large pulmonary embolism, but his coronary arteries were normal and his myocardium showed no sign of ischemia. There is no mention of pathological right to left communication [4].

Livaditis et all published a case of 42 year old woman presenting with painful right leg swelling, which complicated with syncopal episode, followed by abdominal pain and dyspnea. ECG showed changes suggestive of acute myocardial infarction - sinus tachycardia, ST elevations in V1-V3, S wave and ST depression in I, ST depression II, aVL, V5-V6 and ST elevation in aVR. Pulmonary embolism was verified using contrast enhanced CT scan. Shortly after heparin administration ECG changes resolved. Coronarography showed normal coronary arteries [15].

Haghi et all described a case of pulmonary embolism in 61 year old woman, complicated with non-ST segment elevation myocardial infarction (NSTEMI) caused by paradoxical embolism to coronary artery. Cause of hospital admission was shortness of breath and chest pain. ECG showed normal sinus rhythm with RBBB and inverted T waves in leads V1-V4, consistent with NSTEMI. Using contrast enhanced CT they confirmed pulmonary embolism. Coronarography was performed and showed occlusion of first marginal branch in otherwise normal coronary arteries. PFO was confirmed using contrast enhanced transesophageal echocardiography [16].

ECG changes in our case were suggestive of an acute antero-septal myocardial infarction. Most of ECG abnormalities described in presence of pulmonary embolism are of low sensitivity and specificity [3]. ST segment elevation in precordial leads V1-V4 has been reported in few case reports of pulmonary embolism, but exact mechanism of this ECG changes remained unclear [4,5,14,15]. Paradoxical embolism was suggested as possible mechanism but without firm evidence [5,6]. Beside paradoxical embolism to coronary artery two other theories have been suggested as explanation for ST segment elevations in pulmonary embolism. First suggests that sudden pressure load on right ventricle can cause focal or global myocardial ischemia, which can trigger epicardial or microvascular coronary vasospasm, resulting in ST elevation. Second suggests that severe hypoxemia induces a catecholamine surge, which increases myocardial workload and results in ischemia [5].

Diagnosis of paradoxical embolism can only be considered when there is evidence of arterial embolisation in the absence of source in the left heart, source of embolism in venous system and abnormal communication between venous and arterial system. Paradoxical embolism can be considered proven when all of the above statements are meet and there is a thrombus lodged in the abnormal communication between venous and arterial system [16,17]. Most of cases discussed previously lack diagnostic confirmation of abnormal communication between venous and arterial system. Absence of such communication would exclude possibility of paradoxical embolism. Intact coronary arteries are not sufficient proof against paradoxical embolism, since emboli could dissolve by the time of diagnostic procedure.

Conus coronary artery is usually first branch of right coronary artery, but can arise from a separate ostium from right aortic sinus in 33-50% of individuals. Normally it supplies infundibular myocardium of the right ventricular outflow tract and sometimes greater part of anterior wall of the right ventricle [18]. Conus artery occlusion can present as ST segment elevation in precordial leads V1-V3 [19].

Transthoracic echocardiography is an invaluable tool in diagnosis and management of patients with medical emergencies like myocardial infarction, pulmonary embolism and aortic dissection. Transesophageal echocardiography can provide additional information and immediate bedside diagnosis in case of massive pulmonary embolism [20,21]. It is a bedside diagnostic procedure and no transportation out of the ICU is necessary. In case of shocked patient it even seems reasonable to bypass transthoracic echocardiography, in order to save time and hasten establishment of definitive diagnosis and treatment [21]. Transesophageal echocardiography enables diagnosis of right to left intar-atrial shunt in patients with pulmonary embolism, which is associated with increased mortality in patients with paradoxical embolism, and it even predicts lysibility of trombi [22-25].

Ultrasound of lower extremity veins reveling deep venous thrombosis additionally increases clinical certainly in patients with suspected pulmonary embolism [26].

Despite PFO being the most common congenital abnormality in adults that can be present in up to 30% of individuals and 2.9 fold increased relative risk of stroke in first year after pulmonary embolism in patients with PFO, optimal therapy for stroke prevention is still not defined [27].

## Conclusion

Pulmonary embolism is often misdiagnosed due to its varying clinical presentation. It can mimic acute myocardial infarction and can even cause coronary artery obstruction with paradoxical thrombemboli. Transesophageal echocardiography is an important bedside tool in quick diagnosis of pulmonary embolism. Paradoxical embolism to coronary artery can cause ST segment elevations in ECG which are uncommon way of pulmonary embolism presentation. In association with right ventricular ischemia due to increased afterload, paradoxical coronary artery thrombembolism can be another explanation for elevated troponin values and indicates greater morbidity and mortality.

## Competing interests

The authors declare that they have no competing interests.

## Authors' contributions

**TG: **carried out interpretation, drafted manuscript, approved the final manuscript

**MP: **treated the patient, made acquisition of data, carried out interpretation, drafted manuscript, approved the final manuscript

## Supplementary Material

Additional file 1 **Coronaro-angiography with aortography showing occluded conus artery**. Ao- aorta, con- conus artery, RCA- right coronary artery Click here for file

Additional file 2 **Transesophageal echocardiography showing trombemboli in right pulmonary artery**. Tromboemboli (TE) in right pulmonary artery (RPA). Click here for file

Additional file 3 **Transesophageal echocardiography showing right to left intra-atrial shunt after contrast application**. Ao- aorta, LA- left atria, RA- right atria Click here for file
